# Possible Roles of Membrane Trafficking Components for Lipid Droplet Dynamics in Higher Plants and Green Algae

**DOI:** 10.3389/fpls.2019.00207

**Published:** 2019-02-25

**Authors:** Shuxian Huang, Liwen Jiang, Xiaohong Zhuang

**Affiliations:** ^1^Centre for Cell and Developmental Biology, State Key Laboratory of Agrobiotechnology, School of Life Sciences, The Chinese University of Hong Kong, Shatin, Hong Kong; ^2^The Chinese University of Hong Kong Shenzhen Research Institute, Shenzhen, China

**Keywords:** lipid droplet, membrane trafficking, small GTPase, SNARE, ESCRT, retromer, lipophagy

## Abstract

Lipid droplets are ubiquitous dynamic organelles that contain neutral lipids surrounded by a phospholipid monolayer. They can store and supply lipids for energy metabolism and membrane synthesis. In addition, protein transport and lipid exchange often occur between LDs and various organelles to control lipid homeostasis in response to multiple stress responses and cellular signaling. In recent years, multiple membrane trafficking proteins have been identified through LD proteomics and genetic analyses. These membrane trafficking machineries are emerging as critical regulators to function in different LD-organelle interactions, e.g., for LD dynamics, biogenesis and turnover. In this review, we will summarize recent advances in regard to LD-related membrane trafficking proteins and discuss future investigations in higher plants and green algae.

## Introduction

Lipid droplets, which contain neutral lipids, serve as central storage organelles in eukaryotic cells to provide carbon and energy in metabolism, as well as lipids for membrane biosynthesis (Huang, 2018). While our current knowledge on the biogenesis and function of LDs in plants mainly comes from studies on oilseeds, evidence suggests that LDs also play essential roles in various physiological processes and different cell types in plants (e.g., leaves) (Pyc et al., 2017b). Green algae also accumulate considerable amounts of LDs under nitrogen deprivation or stress conditions (Tsai et al., 2015). Moreover, accumulation of LDs under nitrogen starvation is also observed in leaves in higher plant (Brocard et al., 2017). In addition, in both higher plants and green algae, a specific type of LDs called plastoglobuli are formed on the thylakoid membranes in plastids and have been suggested to function in response to oxidative stress or developmental transitions (Van Wijk and Kessler, 2017). Moreover, it has been observed that certain LDs carrying specific enzymes are recruited by the microdomains or lipid rafts in the plasmodesmata (PD) for pathogen defense or cell-to-cell signaling (Van Der Schoot et al., 2011).

**FIGURE 1:**
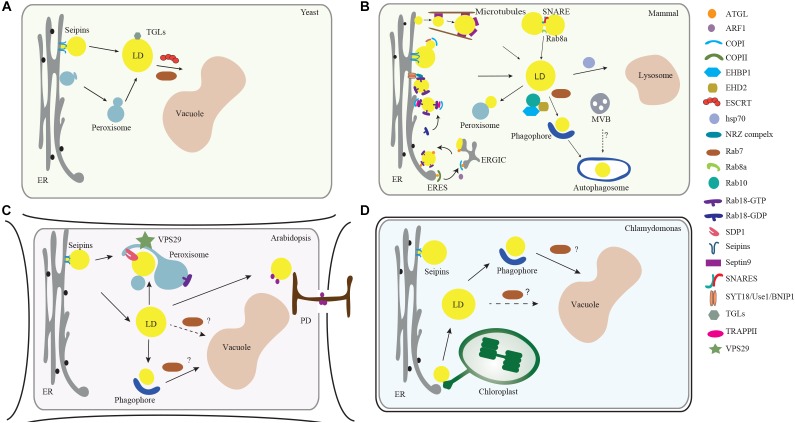
Membrane trafficking regulators in LD dynamics, biogenesis, and turnover in model organisms. **(A)** In yeast, the ESCRT machinery regulates the LDs turnover via microlipophagy (Oku et al., 2017). In addition, Rab GTPase RAB7-like Ypt7 might function together with the HOPS (homotypic fusion and vacuole protein sorting) complex to mediate the fusion of LD with vacuole (Bouchez et al., 2015). **(B)** In mammalian cells, the ER serves as a major platform for LD biogenesis. ARF1 coordinates with COPI vesicles to deliver essential enzymes to the LD surface, while COPI subunits interact with the TRAPPII complex for its recruitment onto LD surface, which functions as a GEF (guanine nucleotide exchange factor) to activate Rab18. Rab18 also forms a complex with the ER-associated proteins NAG-RINT1-ZW10 (NRZ) and SNARE proteins (Syntaxin18, Use1, BNIP1) to control LD growth and maturation (Xu et al., 2018). Rab8 could activate Fsp27 to further promote LD fusion (Wu et al., 2014). In addition, Septin9, a filament-forming cytoskeletal GTPase, might cooperate with the microtubules to regulate LD growth (Akil et al., 2016). For the lipolysis-dependent LD turnover, both COPI and COPII vesicles mediate the delivery of the major lipase ATGL (Soni et al., 2009). During macrolipophagy-mediated LD turnover, LD is sequestered by a preautophagosome structure named phagophore, which will expand and become an autophagosome. Rab10-EHBP1-EHD2 complex as well as Rab7, are implicated to mediate the fusion between LD with MVB, autophagosomes or lysosomes (Schroeder et al., 2015). **(C)** In Arabidopsis plants, VPS29, a retromer subunit, has been shown to function in mediating the trafficking of SUGAR-DEPENDENT 1 (SDP1) from peroxisome to LD surface to regulate both the size and number of LDs through peroxisome tubulation (Thazar-Poulota et al., 2015). Microlipophagy-like process is also reported in plants (Poxleitner et al., 2006). LDs may also contact with the PD to deliver the specific enzymes to the cell wall (Van Der Schoot et al., 2011). Microautophagy is involved in LD turnover but the regulators in mediating the autophagosome-mediated macrolipophagy are unexplored (dashed line, question mark). **(D)** In the green alga *Chlamydomonas*, it is observed that LD formation is highly induced under stress conditions and might be initiated from the ER-chloroplast contact site (Fan et al., 2011). Similar to higher plants, it is reported that LD might be sequestered to the vacuole via microautophagy (Zhao et al., 2014). But how macrolipophagy occurs in this microalgae remains unclear (dashed line, question mark). Abbreviations: ER, endoplasmic reticulum; MVB, multivesicular bodies; LD, lipid droplet; PD, plasmodesmata.

The mechanism of LD biogenesis is conserved to a certain extent among yeast, mammals, higher plants and green algae. For example, the endoplasmic reticulum (ER), where enzymes for neutral lipid synthesis reside to promote LD formation and budding, also serve as a major platform for nascent LD production (Figure 1; Walther et al., 2017). Once LDs are released into the cytoplasm, they display variations in shape, size, and mobility in different plant cell types, developmental stages, and during environmental stress (Pyc et al., 2017b). In addition, to facilitate material exchange and supply, LDs build tight connections with other organelles (e.g., ER, plastids, and vacuoles) via direct membrane contact sites, or vesicle transport, which are largely depends on membrane trafficking machinery (Gao and Goodman, 2015).

In recent years, increasing evidence from both yeast and mammalian cells implies that various membrane trafficking machineries play crucial roles in LD dynamics. However, the underlying molecular mechanisms for regulating LD dynamics in both higher plants and green algae are still not understood. Given the conserved roles of membrane trafficking machineries in endomembrane trafficking in eukaryotes, the working models derived from studies in yeast and mammalian cells will be important for future studies on LDs in higher plants and green algae. In this review, we summarize recent findings in the regulation of LD dynamics with a focus on the related membrane trafficking regulators, as well as in areas needing investigations in plants and green algae in the future.

### LD-Associated Proteins in Higher Plants and Green Algae

The surface of LDs is decorated by the LD-associated proteins, such as oleosin (Huang, 2018). Oleosin, which is unique to higher plants and green algae, is the most abundant structural phospholipid protein found in plant seeds (Huang, 2018). With a long hairpin-like hydrophobic structure, oleosins are able to stabilize LD by inserting into the triacylglyceride (TAG) matrix, so that their hydrophilic N- and C- termini face toward the cytoplasm. Of note, mutations of oleosin can redirect the LD to the ER lumen as well as vacuoles (Huang and Huang, 2017), suggesting that oleosin might be associated with membrane trafficking regulator(s) to control LD relocalization. Recently, it has been shown that a protein family named as lipid droplet-associated proteins (LDAPs), which share high similarity to small rubber particle proteins (SRPPs) in rubber-producing plants, are abundant in non-seed cell types and might play a role in drought stress in Arabidopsis (Gidda et al., 2016; Kim et al., 2016). Through a yeast two hybrid screen, it has been shown that LDAP3 interacts with a plant-specific protein containing an amphipathic α-helix type LD targeting signal, and suppression of this protein alters LD morphology and increases neutral lipid content in seeds (Pyc et al., 2017a).

Although oleosins first evolved in green algae, it was shown that oleosins were only detected in *Chlamydomonas* cells at a certain developmental stage (Huang et al., 2013). By proteomic analysis, a distinct LD structural protein named Major LD Protein (MLDP), which lacks a long hydrophobic tail, has been identified. It was shown that MLDP regulates both the size and number of LDs, as well as TAG degradation. MLDP is not directly distributed on LDs but associated with ER subdomains in close proximity to LDs (Huang et al., 2013), possibly serving the recruitment of different proteins to LDs, like tubulins (Tsai et al., 2015).

In mammalian cells, PLINs (periplins), which is PAT (perilipin/ADRP/TIP47) family protein, represent the major LD-associated protein on the LD surface (Kimmel et al., 2010; Sztalryd and Kimmel, 2014). Although perilipins are less hydrophobic than oleosins, they perform a similar function by inserting into the phospholipid leaflet for binding to neutral lipids. PLINs might also function in protection of LDs from lipolysis, recruitment of lipases and interaction with mitochondria (Sztalryd and Kimmel, 2014). It has been suggested that PLINs are specific to mammalian cells, but a recent study showed that Pet10p, a protein previously identified in yeast LD proteomes, also contains a PAT domain and functions as a yeast perilipin (Gao Q. et al., 2017). Arabidopsis oleosin1 fusion proteins are also targeted to yeast LDs, but complementation assays show that only human PLIN2/3, but not plant oleosins, can restore the *pet10p* mutant. An interaction analysis has demonstrated that Pet10p binds to seipin, an LD assembly factor found on the ER and LD junctions. There are three isoforms of seipin in Arabidopsis, and one in *Chlamydomonas* (Cai et al., 2015; Lu et al., 2017). Of note, in Arabidopsis, SEIPIN1, and SEIPIN2/3 appear to regulate LD size and number with a unique tissue specificity, as SEIPIN1 is exclusively detected in the embryo, while SEIPIN2 and SEIPIN3 display a higher expression pattern in embryos and pollen (Cai et al., 2015; Lu et al., 2017; Taurino et al., 2018). Whether seipin functions in LD biogenesis in *Chlamydomonas* need to be explored.

**Table 1:** Known regulators in LD dynamics and their homologs in Arabidopsis/*Chlamydomonas*.^∗^

Regulator	Specie	Function	Plant (At)	Microalgae (Cr)
**ESCRT**				
VPS27 (ESCRT-0)	Yeast	Regulates the delivery of LDs into the vacuole	N.I.	N.I.
VPS4 (ESCRT-III)	Yeast	Regulates the delivery of LDs into the vacuole	SKD1/VPS4 (At2g27600)	Cre02.g079300
**RAB**				
Rab1	Mammal	Functions in LDs metabolism	RABD2a (At1g02130) RABD2b (At5g47200) RABD2c (At4g17530)	Cre12.g560150
Rab5	Mammal	Functions in LDs metabolism	RABF2a (At5g45130) RABF2b (At4g19640) RABF1 (At3g54840)	Cre12.g517400
Rab7	Mammal	Promotes the direct interactions between MVBs/lysosomes and LDs	RABG3a (At4g09720) RABG3b (At1g22740) RABG3c (At3g16100) RABG3d (At1g52280) RABG3e (At1g49300) RABG3f (At3g18820)	Cre06.g311900
Rab8a	Mammal	Mediates the LDs fusion	RABE1a (At3g53610) RABE1b (At4g20360) RABE1c (At3g46060) RABE1d (At5g03520) RABE1e (At3g09900)	Cre15.g641800
Rab10	Mammal	Mediates the autophagic engulfment of lipid droplets	N.I.	N.I.
Rab18	Mammal	Localizes on the LDs to promote LD formation and turnover	RABC1 (At1g43890) RABC2a (At5g03530) RABC2b (At3g09910)	Cre09.g386900
**SNARE**				
SNAP23	Mammal	Mediates LD fusion	N.I.	N.I.
VAMP4	Mammal	Mediates LD fusion	N.I.	N.I.
Syntaxin-5	Mammal	Mediates LD fusion	SYP31 (At5g05760) SYP32 (At3g24350)	Cre16.g692050
Syntaxin-17	Mammal	Regulates LD biogenesis	N.I.	N.I.
Syntaxin-18	Mammal	Mediates the LD growth and maturation	SYP81 (At1g51740)	Cre17.g711450
**ARFs**				
ARF1	Mammal	Mediates the deliver of ATGL to LDs surface for lipolysis.	ARFA1a (At1g23490) ARFA1b (At5g14670) ARFA1c (At2g47170) ARFA1d (At1g70490) ARFA1e (At3g62290) ARFA1f (At1g10630) ARFB1a (At2g15310) ARFB1b (At5g17060) ARFB1c (A3g03120) ARFC1 (At3g22950) ARFD1a (At1g02440) ARFD1b (At1g02430)	Cre10.g448200
**Retromer**				
VPS29	Arabidopsis	Mediates peroxisome tubulation to deliver the lipase SDP1 to LDs surface	VPS29 (At3g47810)	Cre12.g499900
**Others**				
Septin9	Mammal	Mediate LD accumulation	N.I.	Cre12.g556250

*^∗^Not all the regulators are listed. Abbreviations: At, Arabidopsis thaliana; Cr, *Chlamydomonas reinhardtii*; N.I., not identified. The gene IDs were searched manually at the Arabidopsis Information Resource (TAIR, https://www.arabidopsis.org/) and the Joint Genome Institute (JGI, http://www.jgi.doe.gov/).*

## Membrane Trafficking Regulators in Mediating Ld Dynamics

Two types of degradation pathways: lipolysis and lipophagy, have been reported to regulate LD turnover (Figure 1). Lipolysis requires a set of lipases, but in the latter, LDs are delivered into the vacuole for degradation by either sequestration into a double membrane structure named an autophagosome (macrolipohagy), or by direct engulfment into the lysosome/vacuole lumen (microlipophagy) (Zechner et al., 2017). To perform these two processes, LDs require vesicle transport of the lipases onto the LD surface, and fusion with different compartments (e.g., autophagosomes and vacuole/lysosomes). Several studies have identified multiple membrane trafficking regulators, like small GTPases, as being associated with LDs in LD proteomic analyses from mammalian cells (Brasaemle et al., 2004; Liu et al., 2004). In Arabidopsis and *Chlamydomonas*, several proteins involved in vesicle trafficking and transport were also found by LD proteomic analysis (Nguyen et al., 2011; Zhi et al., 2017). These membrane trafficking machineries are highly conserved in most eukaryotic cells, and their possible roles in LD biogenesis are now being unraveled as discussed in more detail below (Figure 1 and Table 1).

## Small Gtpases in Membrane Tethering Between Ld and Other Organelles

Small GTPases are proteins that regulate membrane trafficking events by recruiting downstream effectors to promote membrane tethering and fusion. Stable connections between LDs and other organelles (e.g., ER, peroxisome, and mitochondria) have been observed in different systems (Gao and Goodman, 2015), and it is reasonable that small GTPases participate in the membrane fusion steps for LDs with other organelles, although the functional roles of these LD contacts are still obscure.

In plants, Rab GTPases have been extensively investigated and reported to function in multiple trafficking pathways (Nielsen et al., 2008). Rab7 proteins are well-known to function in endosome to vacuole trafficking. Thus, overexpression of a Rab7 dominant negative mutant leads to the formation of enlarged PVCs (prevacuolar compartments), fragmented vacuoles, as well as suppression of vacuolar trafficking (Cui et al., 2014). Moreover, malfunctioning of Rab7 also disturbs autophagy by blocking the fusion between autophagosomes and the vacuole membrane (Kwon et al., 2013). In mammalian cells, Rab7 knockdown, or overexpression of the Rab7 mutant form disrupts LD turnover (Liu et al., 2007). Moreover, under starvation conditions, it has been observed that Rab7 is recruited onto the LD surface, which subsequently mediates the docking of endosomes, autophagosomes and lysosomes to the LD surface to promote its degradation via lipophagy (Schroeder et al., 2015). In yeast, the Rab7-like Ypt7p also regulates LD dynamics together with the HOPS complex and V1-ATPase (Bouchez et al., 2015). In Arabidopsis, it has been shown that both oleosin and caleosin are surrounded by a tonoplast marker *α*-TIP during seed germination, suggesting that LDs could be engulfed by the vacuole (Poxleitner et al., 2006). In green algae, LDs are highly induced under nitrogen starvation and might be degraded by autophagy. For instance, LDs are engulfed by the vacuole through a microlipophagy-like process that is observed in heterotrophic cells in *Auxenochlorella protothecoides* (Zhao et al., 2014). In *Micrasterias denticulata*, plastid lipid bodies are sequestered by autophagosome-like structures and delivered to the vacuole for degradation (Schwarz et al., 2017). In *Chlamydomonas*, a set of Rab GTPases has been detected in the secreted ectosomes, including a single copy Rab7 (Long et al., 2016), implying a fundamental role of Rab7 in vesicle transport.

Apart from Rab7, other Rab proteins have been shown to be tightly associated with LDs in mammalian cells, like Rab10, and Rab18. Rab10 recruits adaptor proteins, EHBP1, and EHD2, to form a Rab10-EHBP1-EHD2 complex to promote LD sequestration into the autophagosome, while knockdown of Rab10 or overexpression of its negative mutant form causes a significant accumulation of LDs (Li et al., 2016; Xu et al., 2018). Particularly, Rab18 is the only GTPase that exclusively localizes on the LD surface, and can form distinct complexes either with the ER-associated NAG-RINT1-ZW10 (NRZ) complex or SNARE proteins (Syntaxin18, Use1, BNIP1) in mammalian cells (Martin et al., 2005; Xu et al., 2018). Moreover, activation and recruitment of Rab18 onto the LD surface are mediated by a TRAPPII complex, a Rab GEF (guanine nucleotide exchange factor) that binds to the COPI subunit (Li et al., 2017). On the other hand, Rab8 might promote LD fusion by activation of Fsp27 (Fat-specific protein), which is enriched at the LD-LD contact site for lipid transfer from smaller to larger LDs (Wu et al., 2014). Interestingly, it has been suggested that Rab8, Rab10, and Rab18 are also associated with peroxisomes (Gronemeyer et al., 2013). Except for Rab10, homologs of Rab8 and Rab18 can be found in the Arabidopsis and *Chlamydomonas* genomes (Table 1). Similarly, homologs of Rab8 (RABE1d) and Rab18 (RabC2a) in Arabidopsis are also associated with peroxisome (Hashimoto et al., 2008; Cui et al., 2013). Moreover, RABE1d functions in the pathogen defense response (Camacho et al., 2009), whereas RabC2a interacts with myosin XI (MYA2), which is required for rapid trafficking of peroxisomes (Hashimoto et al., 2005, 2008). Delivery of lipases from peroxisomal extensions to LDs has been observed in Arabidopsis (Thazar-Poulota et al., 2015). Therefore, there is a possibility that Rab8 and Rab18 might function in LD-peroxisome contact in Arabidopsis.

In mammalian cells, other small GTPases like ARF1 and SAR1 have also been implicated in regulating LD homeostasis, by collaboration with COPI and COPII vesicles, respectively, for delivery of lipases (e.g., ATGL) onto LD surfaces for LD turnover (Soni et al., 2009). In mammalian cells, it has been shown that Arf1, together with COPI, are recruited from the cytosol onto the LD surface for the induction of nano-LDs (about 60–80 nm diameter), which may bridge with ER for the recruitment of lipid synthesis enzymes (Wilfling et al., 2014; Li et al., 2017). It is also observed that COPI-like vesicles have interaction with LD in plants (Brocard et al., 2017). In plants, SAR1 and ARF1, as well as motor proteins like myosin, have also been detected in lipidomic and proteomic analyses of isolated LDs from mesocarp and seed kernels (Zhi et al., 2017). In *Chlamydomonas*, it has been suggested that lipid accumulation is also tightly correlated with COPII proteins (Valledor et al., 2014). Recently, it was reported that Septin9, a filament forming related GTPase, might cooperate together with microtubules to regulate LD growth in mammalian cells (Akil et al., 2016). However, SEPTIN can only be found in green algae but not in higher plants. Future efforts are required to validate the possible roles of these different small GTPases in LD dynamics.

## Snare Proteins in Mediating Membrane Tethering During Ld Growth and Maturation

Small GTPases are often linked by distinct effectors to the membrane fusion machinery, indicating that the canonical fusion machinery is also involved in LD fusion. In Arabidopsis, 65 SNAREs have been identified, which are further divided into two groups, the Q-SNAREs with a conserved glutamine residue in the SNARE domain and the R-SNAREs with a conserved arginine residue in the SNARE domain (Bassham and Blatt, 2008). Subcellular localization analysis has revealed that these SNAREs are located on specific intracellular compartments, ranging from the ER, Golgi apparatus, *trans*-Golgi network, endosomes, plasma membrane, PVC to vacuoles (Uemura et al., 2004).

Although little evidence is available that points to SNAREs function in LD dynamics in higher plants and green algae, a growing body of data demonstrates the crucial roles of SNAREs in endomembrane trafficking events. Some plant SNAREs appear to be distributed on more than a single organelle, suggesting an ability to transfer between distinct compartments by paring with other SNAREs (Fujiwara et al., 2014). In support of this, a recent study has uncovered that two distinct sets of SNAREs and Rabs complexes are involved in membrane fusion for endosome-vacuolar and vacuole-vacuole in Arabidopsis, respectively (Takemoto et al., 2018). In a recent proteomics study, two SNARE proteins, AT4G04910, and AT3G56190, have been identified in the isolated LD proteome analysis (Brocard et al., 2017). In *Marchantia polymorpha*, it has been observed that two Q-SNAREs, MpSYP13A, and MpSYP12B, localize to the oil body membrane (Kanazawa et al., 2016). It was also reported that two SNARE proteins (Vmpl2 and Vamp74) in *Chlamydomonas* are highly induced during autophagy (Ramundo and Rochaix, 2014). Particularly, *Chlamydomonas* cells lacking CrVMP1, which contains a predicted SNARE domain, exhibit defects in macroautophagy and TAG metabolism, indicating a possible role of CrVMP1 in autophagy-mediated LD degradation (Tenenboim et al., 2014). Indeed, a VMP1 homolog in mammalian cells functions in ER-LD contacts to contribute to autophagosome formation (Zhao et al., 2017). Future studies should provide novel insights by investigating how these SNAREs are targeted to LDs and regulate LD-organelle contacts.

Interestingly, previous studies in mammalian cells have shown that LD might sequester a novel v-/t-SNARE complex containing SNAP23, syntaxin-5, and VAMP4, to regulate the exocytosis of glucose transporters (Bostrom et al., 2007). Knockdown of SNAP23, syntaxin-5, or VAMP4, all suppress the fusion and impair the size of LDs. In addition, it has been shown that knockdown of SNAP23 interrupts the interaction between mitochondria and LD (Jagerstrom et al., 2009). LD-mitochondrial contacts have been implicated to mediate the direct flow of fatty acids from LD to mitochondria for further oxidation to release energy (Gao and Goodman, 2015). Moreover, SNAP23 also collaborates with Syntaxin17 to regulate the distribution of the ACSL3 (acyl-CoA synthetase 3), an important enzyme localized on the ER for LD biogenesis (Kimura et al., 2018). A recent study showed that another SNARE protein, Syntaxin18, works together with the Rab18 and NRZ (NAG-RINT1-ZW10) to establish a direct contact between LD and ER allowing LD growth and maturation (Xu et al., 2018). These studies all support the notion that LDs employ different combinations of SNAREs to mediate LD tethering with distinct compartments.

## The Escrt Machinery in Mediating the Ld Degradation

The ESCRT machinery, which is conserved in eukaryotic cells, is essential for the scission of narrow membrane necks during the budding of intraluminal vesicles (ILVs) into the multivesicular body (MVB) (Gao C. et al., 2017). Recently, yeast ESCRT components have also been demonstrated to function in LD turnover during microautophagy, although independent of the core Autophagy-related (ATG) genes (Oku et al., 2017). It has been shown that yeast Vps27 may interact with clathrin and translocate into the vacuole after diauxic shift, which leads to the incorporation of LDs into the vacuole for LD degradation. In addition, mutation in Vps4, which functions at the final step for ESCRT disassembly, leads to a failure in LD degradation (Vevea et al., 2015). In a recent study by genetic screening, several ESCRTIII deletion mutants have been identified, displaying defects in LD metabolism in a lipophagy independent manner (Mast et al., 2018). With its membrane scission ability, it is very likely that the ESCRT machinery may directly induce the invagination of LD by the vacuolar membrane.

In plants, most ESCRT homologs play conserved roles in ILV formation and MVB biogenesis (Gao C. et al., 2017). Plant unique ESCRT components such as FYVE DOMAIN PROTEIN REQUIRED FOR ENDOSOMAL SORTING 1 (FREE1) and POSITIVE REGULATOR OF SKD1 (PROS) have also been recently identified (Gao C. et al., 2014; Reyes et al., 2014). Recent studies have implied that FREE1 might play a dual role in regulating autophagosome-vacuole fusion and vacuole biogenesis in Arabidopsis (Gao et al., 2015; Kalinowska et al., 2015). In addition, CHMP1 has also been involved in the autophagic turnover of plastid materials (Spitzer et al., 2015). However, it remains unexplored as to whether the ESCRT components are involved in LD turnover in plants. Of note, several ESCRT proteins have been identified by analyzing the ectosomes released from the flagella lipids in *Chlamydomonas*, and ectosome secretion is disturbed in ESCRT mutants (Long et al., 2016). More investigation is needed to figure out if ESCRT can play a directly role in LD degradation in plants and algae.

## Retromer and Transport Between Ld and Peroxisomes

The retromer complex mediates retrograde transport from the endosome to other compartments (Heucken and Ivanov, 2018). In Arabidopsis, the retromer complex consists of SNX1, SNX2a/b, VPS35a/b/c, VPS26a/b, and VPS29 (Heucken and Ivanov, 2018). Intriguingly, a recent study has uncovered a novel function of retromer in LD homeostasis. At the early stage of seed germination, SDP1 (SUGAR-DEPENDENT 1), the major lipase for LD degradation, is localized on the peroxisome, and then transported to the LD surface via peroxisome tubulation (Thazar-Poulota et al., 2015). However, in *vps29* mutant, the formation of the SDP1 peroxisomal tubulations is inhibited, resulting in a suppression in both SDP1 migration and lipid mobilization. The molecular mechanism of retromer-mediated peroxisomal tubulation in LD homeostasis remains to be elucidated, and it is possible that retromer functions in recycling of other regulators from peroxisomes. Particularly, the role of retromer in LD turnover has so far only been reported in plants, and whether it also operates in other eukaryotes is unclear.

## Future Perspectives

Emerging findings have greatly expanded our current knowledge about the crucial roles of LDs in eukaryotic cells. While most of the work has focussed on the characterization of physiological roles of LDs in higher plants and green algae, the underlying mechanism of LD homeostasis is still unclear. In regard to the complex cross-talk between LDs and other compartments, more attention should be given to a molecular understanding of LD dynamics mediated by membrane trafficking systems. Future studies with genetic screens, proteomics, as well as mechanistic studies of the candidate proteins will shed new light on this field. We anticipate that these basic studies underlying lipid droplet dynamics would provide useful tools to be applied in plant stress response, seed oil production and biofuels.

## Author Contributions

XZ designed the concept and the organization of the manuscript. All authors wrote the manuscript. XZ and LJ edited the manuscript.

## Conflict of Interest Statement

The authors declare that the research was conducted in the absence of any commercial or financial relationships that could be construed as a potential conflict of interest.
